# Ribo-uORF: a comprehensive data resource of upstream open reading frames (uORFs) based on ribosome profiling

**DOI:** 10.1093/nar/gkac1094

**Published:** 2022-11-28

**Authors:** Qi Liu, Xin Peng, Mengyuan Shen, Qian Qian, Junlian Xing, Chen Li, Richard I Gregory

**Affiliations:** Rice Research Institute, Guangdong Academy of Agricultural Sciences, Guangzhou 510640, China; Guangdong Key Laboratory of New Technology in Rice Breeding, Guangzhou 510640, China; Guangdong Rice Engineering Laboratory, Guangzhou 510640, China; Key Laboratory of Genetics and Breeding of High Quality Rice in Southern China (Co-construction by Ministry and Province), Guangzhou 510640, China; Rice Research Institute, Guangdong Academy of Agricultural Sciences, Guangzhou 510640, China; Guangdong Key Laboratory of New Technology in Rice Breeding, Guangzhou 510640, China; Guangdong Rice Engineering Laboratory, Guangzhou 510640, China; Key Laboratory of Genetics and Breeding of High Quality Rice in Southern China (Co-construction by Ministry and Province), Guangzhou 510640, China; Rice Research Institute, Guangdong Academy of Agricultural Sciences, Guangzhou 510640, China; Guangdong Key Laboratory of New Technology in Rice Breeding, Guangzhou 510640, China; Guangdong Rice Engineering Laboratory, Guangzhou 510640, China; Key Laboratory of Genetics and Breeding of High Quality Rice in Southern China (Co-construction by Ministry and Province), Guangzhou 510640, China; Rice Research Institute, Guangdong Academy of Agricultural Sciences, Guangzhou 510640, China; Guangdong Key Laboratory of New Technology in Rice Breeding, Guangzhou 510640, China; Guangdong Rice Engineering Laboratory, Guangzhou 510640, China; Key Laboratory of Genetics and Breeding of High Quality Rice in Southern China (Co-construction by Ministry and Province), Guangzhou 510640, China; Rice Research Institute, Guangdong Academy of Agricultural Sciences, Guangzhou 510640, China; Guangdong Key Laboratory of New Technology in Rice Breeding, Guangzhou 510640, China; Guangdong Rice Engineering Laboratory, Guangzhou 510640, China; Key Laboratory of Genetics and Breeding of High Quality Rice in Southern China (Co-construction by Ministry and Province), Guangzhou 510640, China; Rice Research Institute, Guangdong Academy of Agricultural Sciences, Guangzhou 510640, China; Guangdong Key Laboratory of New Technology in Rice Breeding, Guangzhou 510640, China; Guangdong Rice Engineering Laboratory, Guangzhou 510640, China; Key Laboratory of Genetics and Breeding of High Quality Rice in Southern China (Co-construction by Ministry and Province), Guangzhou 510640, China; Stem Cell Program, Division of Hematology/Oncology, Boston Children's Hospital, Boston, MA 02115, USA; Department of Biological Chemistry and Molecular Pharmacology, Harvard Medical School, Boston, MA 02115, USA; Department of Pediatrics, Harvard Medical School, Boston, MA 02115, USA; Harvard Initiative for RNA Medicine, Boston, MA 02115, USA; Harvard Stem Cell Institute, Cambridge, MA 02138, USA

## Abstract

Upstream open reading frames (uORFs) are typically defined as translation sites located within the 5′ untranslated region upstream of the main protein coding sequence (CDS) of messenger RNAs (mRNAs). Although uORFs are prevalent in eukaryotic mRNAs and modulate the translation of downstream CDSs, a comprehensive resource for uORFs is currently lacking. We developed Ribo-uORF (http://rnainformatics.org.cn/RiboUORF) to serve as a comprehensive functional resource for uORF analysis based on ribosome profiling (Ribo-seq) data. Ribo-uORF currently supports six species: human, mouse, rat, zebrafish, fruit fly, and worm. Ribo-uORF includes 501 554 actively translated uORFs and 107 914 upstream translation initiation sites (uTIS), which were identified from 1495 Ribo-seq and 77 quantitative translation initiation sequencing (QTI-seq) datasets, respectively. We also developed mRNAbrowse to visualize items such as uORFs, *cis*-regulatory elements, genetic variations, eQTLs, GWAS-based associations, RNA modifications, and RNA editing. Ribo-uORF provides a very intuitive web interface for conveniently browsing, searching, and visualizing uORF data. Finally, uORFscan and UTR5var were developed in Ribo-uORF to precisely identify uORFs and analyze the influence of genetic mutations on uORFs using user-uploaded datasets. Ribo-uORF should greatly facilitate studies of uORFs and their roles in mRNA translation and posttranscriptional control of gene expression.

## Graphical Abstract

**Graphical Abstract:**
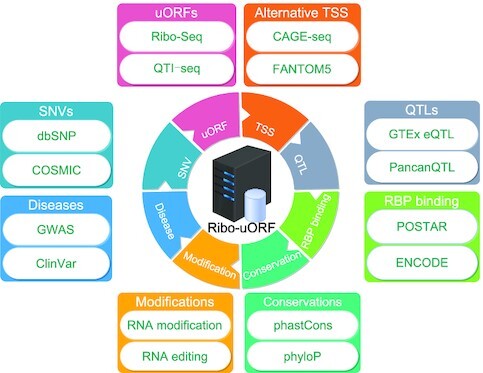
Ribo-uORF is a comprehensive database that provides valuable resources and analysis tools for the identification and characterization of uORFs based on ribosome profiling data and several related resources.

## INTRODUCTION

Gene expression is regulated at both transcriptional and posttranscriptional levels. Posttranscriptional control mechanisms include altered mRNA stability and regulation of mRNA translation. In general, mRNA is composed of translated coding sequences (CDSs) and untranslated regions (UTRs) (1,2). The 5′ UTR, an important mediator of translational regulation, contains many different elements, such as internal ribosome entry sites (IRESs) (3), G-quadruplexes (4,5) and Kozak consensus sequences (6). Upstream open reading frames (uORFs) are defined by a start codon upstream of the canonical translation initiation site and the main open reading frame (mORF), and can be translated into short peptides (7,8). uORFs are divided into three classes: (i) non-overlapping uORFs (that contain stop codons located upstream of AUG start codons of CDSs [mAUGs]), (ii) out-of frame overlapping uORFs (that have stop codons located downstream of mAUGs and are in a different reading frame) and (iii) N-terminal extension uORFs (whereby the uORFs are in-frame and overlap with the mORF and share the same stop codon with the CDS) (9). As evidenced by the detection of certain short peptides by mass spectrometry (MS), many uORFs are translated from non-AUG start codons (10,11). In addition, numerous mRNA modifications reportedly occur in 5′ UTRs, including *N*^6^-methyladenosine (m^6^A), *N*^1^-methyladenosine (m^1^A) and *N*^7^-methylguanosine (m^7^G); among these, the dynamic regulation of m^6^A in uORFs can control the translation of downstream main ORFs (mORFs) (12).

uORFs play a critical role in regulating translation initiation of CDSs (2,13). When a pre-initiation complex (PIC) scans along a uORF-containing 5′ UTR, three scenarios are possible. In the first scenario, normal translation of the downstream mORF is unaffected, as the scanning PIC fails to recognize the start codon of the uORF and continues to search for the next start codon (‘leaky scanning’) (14). The second possibility is that the PIC successfully recognizes the start codon of the uORF, causing mORF translation to be reduced, and the 40S subunit then remains bound to the mRNA and re-initiates translation at the downstream mORF start codon after termination at the uORF stop codon (‘re-initiation’) (15). In the third case, translation of the downstream mORF is suppressed by termination and ribosome drop off at the uORF stop codon, thus triggering nonsense-mediated mRNA decay (‘stall’/‘drop off’) (16). An increasing number of reports of uORFs producing functional short peptides (uORF-encoded peptides, or uPEPs) have also appeared (17,18). For example, a large-scale CRISPR knockout screening identified multiple uPEPs (19). According to one study, uPEPs can act as ligands for MHC class I molecules to elicit T cell responses (20). Previous studies have suggested that nearly 50% of all human and mouse mRNAs contain uORFs, and mutations within uORFs are linked to many diseases (21–24). For example, the insertion of GGC repeats in a uORF of the *NOTCH2NLC* gene can cause neuronal intranuclear inclusion disease (25). uORF-mediated translational suppression of PLK4 has a critical role in the prevention of centriole amplification and preservation of the genomic integrity of future gametes (26). Genetic ablation of uORF initiation codons in tyrosine kinase transcripts enhances translation of associated mORFs in human cells (27). In addition, genome-wide studies over the past several years have revealed the widespread regulatory functions, genetic variation, and evolutionary selection of uORFs in different species in different biological contexts (28–32).

Ribosome profiling (Ribo-seq) based on deep sequencing enables transcriptome-wide and quantitative analysis of uORFs and facilitates potential exploration of regulatory functions of individual uORFs (33–35). Furthermore, convenient pipelines for the quality control and statistical analysis of Ribo-seq data, ensuring high accuracy and levels of confidence, are currently available (36,37). Over the past decade, a large amount of Ribo-seq (∼3000 samples) and quantitative translation initiation sequencing (QTI-seq) (∼100 samples) data has been generated from human and mouse cells/tissues and made publicly available in Gene Expression Omnibus (GEO) and Sequence Read Archive (SRA) databases. Although several uORF-related databases, such as uORFdb (38), TISdb (39), sORFs.org (40) and SmProt (41), already exist, they are quite specific, offer limited functionalities, and/or are not based on the large amount of currently available Ribo-seq data. A comprehensive uORF database based on the extensive quantity of available Ribo-seq data and offering user-friendly analysis tools is therefore urgently needed to help characterize uORF-mediated gene regulation.

In this study, we developed Ribo-uORF (http://rnainformatics.org.cn/RiboUORF or http://rnabioinfor.tch.harvard.edu/RiboUORF), a comprehensive resource for the functional annotation of uORFs. Ribo-uORF includes annotations of *cis*-regulatory elements and GWAS/disease mutations and information on genetic variations, eQTLs, RNA modifications and editing, RBP-binding sites, and uORFs reported from the literature or gleaned from a very large amount of Ribo-seq and QTI-seq data. We also developed uORFscan, an easy-to-use bioinformatics software program, to precisely call and annotate uORFs from Ribo-seq data. Another convenient tool, UTR5var, was constructed for annotating the effect of genetic variants on uORFs. Ribo-uORF also features a very intuitive web interface for simple direct, browsing, searching, and visualizing uORF data, thereby providing a valuable resource and analysis tool for the identification and characterization of uORFs involved in the posttranscriptional control of gene expression.

## MATERIALS AND METHODS

### Ribo-seq datasets and reference genomes

**Figure 1.**
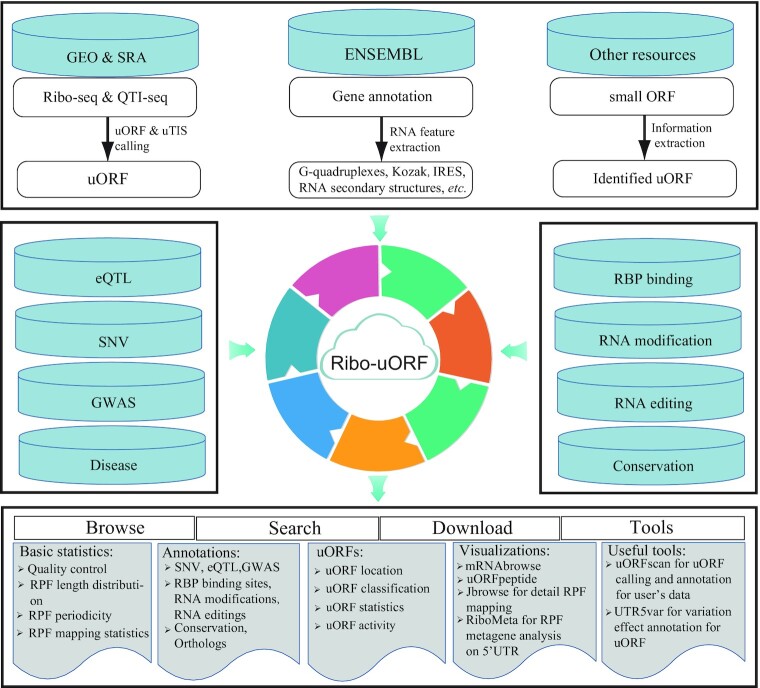
Ribo-uORF workflow overview. Ribo-uORF provides comprehensive information on uORFs (upstream open read frames) generated from 1495 Ribo-seq and 77 QTI-seq datasets. Ribo-uORF integrates an additional 18 related datasets, such as IRES, G-quadruplex secondary structures, eQTL and RNA modification datasets, from sequence annotations and public databases. All results generated by Ribo-uORF are deposited in a MySQL database and are displayed in several convenient modules on web pages. Meanwhile, we developed many convenient tools in Ribo-uORF to facilitate users visualize the data in the database and to identify uORFs from their Ribo-seq data.

We manually collected high-throughput Ribo-seq and QTI-seq data from GEO and SRA databases (Figure 1). Cutadapt v3.4 (42) and FASTX-Toolkit v0.0.13 (http://hannonlab.cshl.edu/fastx_toolkit/) were used for adapter trimming and quality control of raw reads. The resulting clean reads were collapsed into FASTA format using a Perl script. To remove contaminating non-mRNA reads, we identified rRNA, tRNA, and snRNA reads using Bowtie v1.2.2 (43) with default parameters. Next, tRNA sequences were downloaded from the GtRNAdb v2.0 database (44), and rRNA and snRNA sequences were retrieved from noncoding RNA annotations in the Ensembl database (release 103) (45). Reference genomes, including genome sequences, protein-coding sequences, and gene annotations, were downloaded for six species as follows: human (V32 version for hg38) and mouse (M23 version for mm10) from the GENCODE database (46), and rat (rn6), zebrafish (GRCz11), fruit fly (BDGP6), and worm (WBcel235) from the Ensembl Genomes database (release 103) (45). Sequences of the 5′ UTR, defined as the untranslated region between the CDS transcriptional start site and the site of translation initiation, were extracted from the GENCODE database (for human and mouse) and the ENSEMBL database (for other species). The overall Ribo-uORF workflow is shown in Figure 1.

### Data analysis pipeline

Using the ORFannotate.pl program in RibORF (47), we first obtained all candidate ORFs, which are defined as all possible start-to-stop ORFs (7) in transcript sequences based on ATG and the other four most frequently used near-cognate start codons (CTG/GTG/TTG/ACG) and the three stop codons (TAG/TGA/TAA) by ORFannotate.pl program in RibORF (47). The minimum ORF length was set to 18 nucleotides (six amino acids). Meanwhile, all the potential uORFs that entirely overlap with CDSs (main ORFs) were discarded to remove these artificial uORFs. All candidate uORFs were then classified according to transcript type and their location within transcripts into three categories: non-overlapping uORFs, out-of-frame overlapping uORFs and N-terminal extension (in-frame overlapping) uORFs. Non-overlapping uORFs were defined as translated sequences located within the 5′ UTR of annotated protein-coding genes, whereas out-of-frame overlapping uORFs corresponded to translated sequences beginning in the 5′ UTR and partially overlapping the CDS of an annotated protein-coding gene. Finally, N-terminal extension uORFs were considered to be translated sequences beginning in the 5′ UTR and sharing the same stop codon with the annotated CDS of a protein-coding gene. We modified the original ORFannotate.pl program in RibORF to include a function for translating uORFs into peptides, which were then used for signal peptide analyses. To speed up the analysis, we distributed the candidate ORFs into different files according to chromosome. The main RibORF.pl program was modified accordingly, and subsequent analyses were conducted in parallel for each chromosome.

Actively-translated uORFs, which are defined as the translated candidate uORFs supported by Ribo-seq data, were called and analyzed using an integrated pipeline developed and packaged as uORFscan in this study. Given the small size (26–34 nt) of ribosome-protected fragments (RPFs) in the Ribo-seq data, we first converted the input format of RibORF to a collapsed FASTA format (e.g. ‘>seq1_x160’, where ‘seq1’ is the unique ID of a clean RPF, and ‘160’ represents the frequency of RPFs of ‘seq1’), thereby further increasing the mapping speed of the large amount of Ribo-seq data. Next, clean RPFs were mapped to the reference genome using STAR v2.7.4a (48). Unique genome-mapped RPFs were then mapped against protein-coding transcripts using Bowtie v1.2.2 (43). RPF reads mapped to the genome with STAR in end-to-end mode were extracted by removing the soft clipped reads from the BAM file. The RibORF (47) main program was then used to identify ORFs on the basis of 3-nt periodicity distribution and the uniformity of RPF distribution using threshold of pred.pvalue > 0.7. Next, actively translated uORFs were identified and classified with the unified pipeline used for candidate uORFs. In some cases, actively translated uORFs sharing the same stop codon but with different start codons were identified for a given transcript in one Ribo-seq sample. In these cases, we selected the uORF with an AUG start codon closest to the 5′ end of the mRNA as the representative one in this sample. If no RPF was present between the selected codon and the next downstream candidate, however, we chose the uORF with next start codon as the representative uORF in this sample. The uORFs with same genomic coordinates from different isoforms of one gene were also collapsed into unique ones to facilitate the comparison between different species. QTI/GTI-seq data for human and mouse were collected from the SRA database, which contained no data for the other four study organisms. Ribo-TISH v0.2.7 (49) was used to determine all active upstream translation initiation sites (uTIS) with ATG and other four near-cognate start codons in the QTI-seq data, which were used to support confidence in our identification of the translation initiation sites of uORFs.

### Integrated uORF annotation of multiple published datasets

The uORF information retrieved from TISdb (v1.0) (39), sORFs.org (v2.0) (40), uORFdb (38) and Phase I consensus sets of Ribo-seq ORFs from GENCODE (50) was integrated into Ribo-uORF. To investigate relationships between uORFs and disease-related SNVs and SNPs, we downloaded cancer somatic mutations from the COSMIC v93 database (51). The human disease/trait-associated SNPs were curated from published GWAS data provided by the NHGRI GWAS Catalog (all associations v1.0) (52). *Cis*- and *trans*-eQTLs in 33 cancer types were downloaded from PancanQTL (20220416) (53). eQTLs from GTEx v8 (54) were also included in Ribo-uORF. In addition, we collected datasets from REDIportal v2.0 (55) for RNA editing; RMBase v2.0 (56), Met-DB v2.0 (57), REPIC v1.0 (58) and m6A_Atalas v1.0 (59) for RNA modifications; dbSNP v155 (60) and ClinVar (clinvar_20220416) (61) for genetic variations; POSTAR v2 (62) and ENCODE (20220423) (63) for RBP binding sites; Fantom v5 (64) for alternative tag start sites; RNA secondary structures by DMS-seq from RASP v1.0 (65); and phastCons (66) and phylop (67) for sequence conservation. Signal peptide and cleavage sites were analyzed using SignalP v4.1 with default parameters (68). The G-quadruplex propensity of sequences was predicted using G4Hunter (released on 8 April 2020) (69) with default parameters, and IRESfinder v1.1.0 with default parameters was used to identify putative IRESs (internal ribosome entry sites) (70). Annotation of Kozak consensus sequences was based on a motif search using an in-house developed Perl program (strong: [AG]NNAUGG; moderate: [AG]NNAUGN or NNNAUGG; weak: NNNAUGN). All datasets were transformed into BED6 format and then associated with uORF regions using the data.table package in the R platform. The composition of human datasets in the Ribo-uORF database is shown in Supplementary Figure S1.

### Construction of the Ribo-uORF web interface

The database is hosted within the Apache environment under a Linux system equipped with two Octa-core AMD processors (2.6 GHz each) and 64GB of RAM. All datasets were processed and stored in a MySQL database system. The database query and user interface was developed using PHP and JavaScript. The query result table was based on jQueryUI and DataTables, which are highly flexible tools for sorting and filtering search results. Interactive plots were generated using the Highcharts JavaScript library (https://www.highcharts.com). JBrowse (v1.15.4) (71), a web-based genome browser, was selected to construct a browser for uORF visualization on the genomic scale. We developed mRNAbrowse, a tool based on IGV.js (https://github.com/igvteam/igv.js), for transcript-scale visualization of uORFs, *cis*-regulatory elements, genetic variations, eQTLs, GWAS-based associations, RNA modifications and RNA editing and RBP-binding sites. SeqViz (https://github.com/Lattice-Automation/seqviz), a sequence viewer supporting multiple input formats and display settings, was used to develop uORFpeptide for visualization of potential peptides encoded by uORFs.

## DATABASE CONTENT AND WEB INTERFACE

### Comprehensive atlas of uORFs from six species

**Figure 2.**
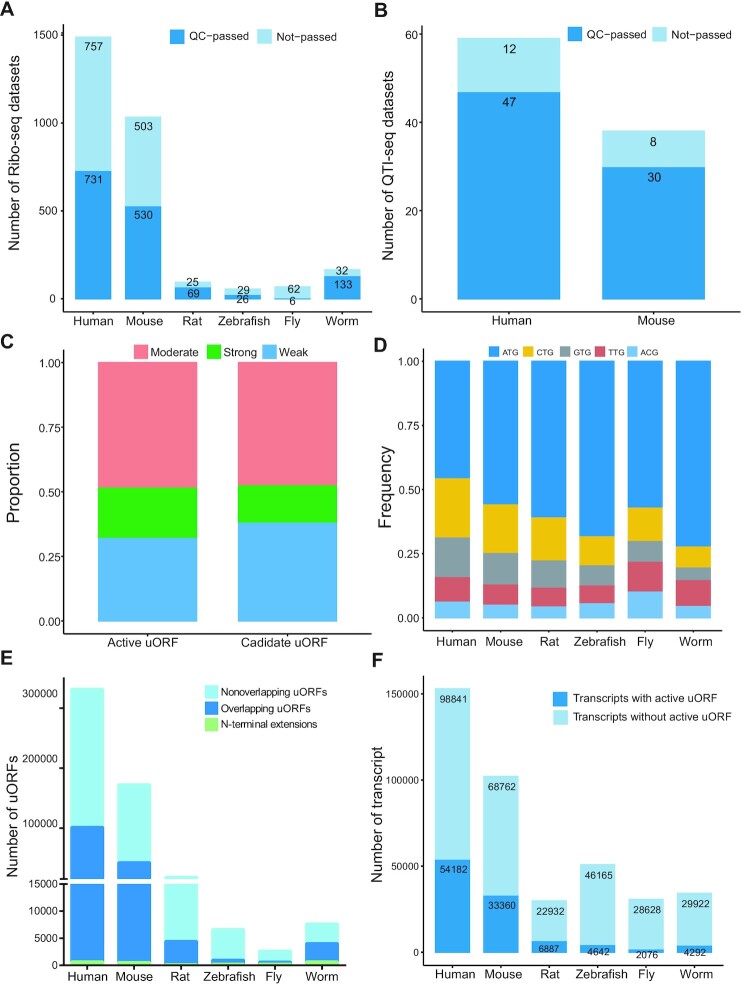
Comprehensive atlas of uORFs from six species. (A, B) The number of remaining samples after quality control (QC) for Ribo-seq (**A**) and QTI-seq (**B**) datasets. Ribo-seq and QTI-seq datasets with a collapsed FASTA file size smaller than 5MB, a RPF peak size distribution outside of the range of 27–32 bp, or having no maximum distribution of RPFs in coding frame 0 or with the percentage of RPFs in frame 0 less than 50% were excluded from uORF identification. (**C**) Statistics on Kozak strengths of active uORFs and candidate uORFs. (**D**) Usage frequencies of start codons of actively translated uORFs. (**E**) The number of actively translated uORFs by category. Non-overlapping, overlapping, and N-terminal extensions respectively refer to non-overlapping uORFs, out-of-frame overlapping uORFs, and N-terminal extension uORFs. (**F**) The number of transcripts with actively translated uORFs.

A total of 2903 Ribo-seq samples were downloaded from GEO and SRA databases, namely, 1488 human (*Homo sapiens*), 1033 mouse (*Mus musculus*), 94 rat (*Rattus norvegicus*), 55 zebrafish (*Danio rerio*), 68 fruit fly (*Drosophila melanogaster*) and 165 worm (*Caenorhabditis elegans*) datasets. Three criteria were used to filter out raw sequencing data of low quality. First, only collapsed FASTA input files larger than 5 Mb were retained, as calling ORFs from smaller Ribo-seq datasets was difficult. The second criterion was that the RPF size distribution should be within 26–34 bp and the size with maximum frequency (the peak) should be within 27–32 bp (Supplementary Figure S2A). Third, datasets in which the maximum distribution of RPFs was not present in coding frame 0 and the percentage of RPFs in coding frame 0 was not larger than 50% were removed. After Ribo-seq quality control, 731 human, 530 mouse, 69 rat, 26 zebrafish, 6 fruit fly and 133 worm datasets were retained for further analysis (Figure 2A and Table S1). We also collected 97 QTI-seq samples, of which 47 and 30, respectively, of the 59 human and 38 mouse datasets passed QTI-seq quality control (Figure 2B and Table S1). After quality control, the remaining Ribo-seq and QTI-seq data were of good quality, as confirmed by the distribution of RPF lengths and a metagene plot produced using uORFscan (Supplementary Figure S2). The Kozak consensus sequence is a nucleic acid motif that plays a major role in the initiation of the translation process (6). Most Kozak sequence strengths were strong or moderate, which suggests that the uORFs identified by Ribo-uORF are actively expressed (Figure 2C). The most prominent start triplet of uORFs in human, mouse, zebrafish, and rat was ‘ATG’, followed in order by ‘CTG’, ‘GTG’, ‘TTG’ and ‘ACG’ (Figure 2D). In fruit fly, the order of usage frequencies of the five start triplets were ATG, CTG, TTG, ACG and GTG. In worm, however, the prominent start triplets followed the order of ‘ATG’, ‘TTG’, ‘CTG’, ‘GTG’ and ‘ACG’ (Figure 2D)

RibORF was used to identify translated ORFs in the clean Ribo-seq reads. In total, we identified 1 183 503, 778 278, 224 207, 478 696, 551 446 and 131 171 candidate uORFs in human, mouse, rat, zebrafish, fruit fly, and worm, respectively (Supplementary Figure S3A). Among the candidate uORFs uncovered by bioinformatic prediction in human and mouse, 25.92% (306 763) and 20.56% (159 999) were respectively supported by Ribo-seq uORFs (Figure 2E). The most abundant uORFs were non-overlapping uORFs, followed by overlapping and N-terminal extension ones (Figure 2E and Supplementary Figure S3A). We detected 51.40% (78 647) and 52.02% (53 126) transcripts with candidate uORFs in human and mouse, respectively, which is consistent with previous results (21,22) (Supplementary Figure S3B). In addition, 35.41%, 32.67%, 23.10%, 9.14%, 6.76% and 12.54% of transcripts in human, mouse, rat, zebrafish, fruit fly and worm, respectively, contained actively translated uORFs that were identified from the candidate uORFs based on RPF 3-nt periodicity and uniformity of read distribution (Figure 2F). The ratios of transcripts with active uORFs may have been influenced by the relative abundance of Ribo-seq datasets and the number of reported isoforms in each species. Using the QTI-seq data, a total of 79 416 and 28 498 uTISs were respectively identified from 47 human and 30 mouse QTI-seq datasets (Supplementary Figure S3C). We found 37 305 and 16 002 mRNAs possessing upstream translation initiation sites (uTISs) in human and mouse, respectively (Supplementary Figure S3D).

### Web-based modules developed to explore uORFs

**Figure 3.**
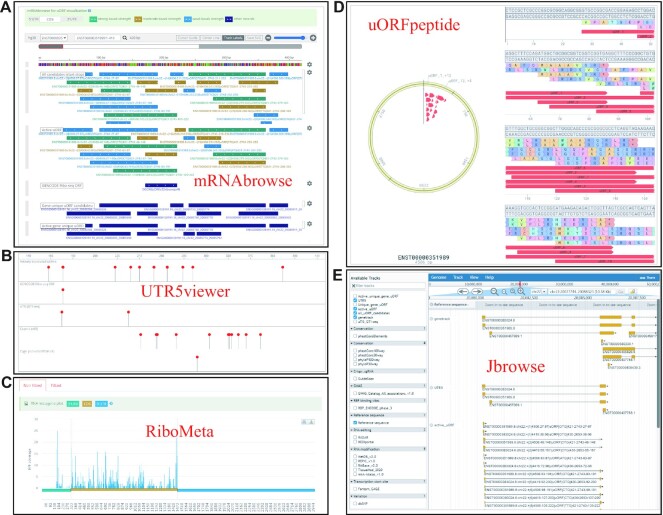
Overview of the Ribo-uORF database visualization modules. Ribo-uORF Transcript Browser module contains mRNAbrowse (**A**) and UTR5viewer (**B**). The mRNAbrowse shows tracks of uORFs and other related annotations, such as eQTLs and RNA modifications, with transcript coordinates. In addition, UTR5viewer was designed to compare the locations of uORFs with other elements in 5’UTR. (**C**) RiboMeta was developed to show metagene plot displaying the distribution of RPFs on 5′ UTRs, CDSs, and 3′ UTRs. (**D**) uORFpeptide were developed to display details of potential encoded peptide sequences of uORFs. (**E**) Jbrowse displaying tracks of uORFs and other related annotations, such as eQTLs and RNA modifications, with genomic coordinates.

We developed convenient web-based modules to help users quickly retrieve uORFs and detailed annotations from Ribo-uORF (Figure 3). In ‘Browse’ module, users can query datasets by species, gene, transcript, and sample (including Ribo-seq and QTI-seq). Each gene or transcript annotation is linked to the GENCODE database (for human and mouse) and the Ensembl Genomes database (for rat, zebrafish, fruit fly, and worm). Sample details of Ribo-seq and QTI-seq data, including basic information from SRA or GEO databases, are also provided in table format in the ‘Browse’ module. Sample details, including quality statistics and a list of uORFs, are displayed by clicking the hyperlink associated with the ‘Results’ column. Users can also click the ‘plus’ button on the right side of the table to obtain more detailed information, such as eQTLs and RNA modifications. When users click the ‘Detail’ icon at the bottom of the screen, detailed features of uORFs are displayed via three intuitive visualization tools developed in the database (Figure 3A, B and D). In particular, locations of all candidate uORFs, actively translated uORFs, and uTISs and also related information, such as GWAS Catalog (52) and REPIC (58), are displayed along with transcript coordinates in mRNAbrowse (Figure 3A). It is noted that Kozak classification of each ORF is used for visualization of the actively translated ORFs and not provided as its own track. The structure score by DMS-seq strategy is shown in a separate track to indicate the pairing probability of each nucleotide, which can represent the density of RNA stem loop structures. At the same time, UTR5viewer and uORFpeptide are automatically loaded to display details of uORF sequences (Figure 3B and D). Furthermore, RiboMeta tool is developed in Ribo-uORF to visualize the metagene distribution of RPFs on transcripts (Figure 3C). It is noted that two visualization modes (histogram model and line model based on loess fitting) are provided in RiboMeta.

Information on supporting samples for transcripts with actively translated uORFs and a link to the metagene page are also provided in the ‘Browse’ module. The metagene plot displays the distribution of RPFs in 3′ UTRs, CDSs, and 5′ UTRs (Figure 3C). Moreover, all uORFs and other publicly available annotations can be visualized using the genome browser in the ‘Jbrowse’ module (Figure 3E). Information and download links for each dataset can be accessed in Ribo-uORF using the ‘Download’ module. In the ‘Search’ module, users can also query the Ribo-uORF datasets by Ensembl ID or gene name.

### Web-based tools developed for user-uploaded Ribo-seq datasets

**Figure 4.**
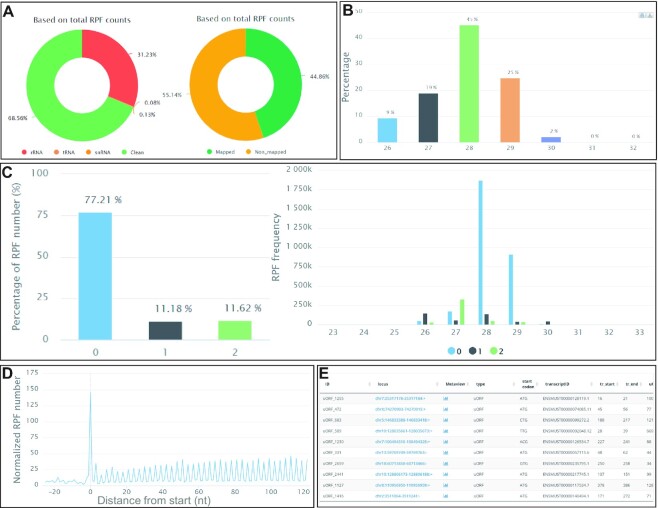
Screenshots of uORFscan outputs. All uORFscan outputs are presented via an intuitive web interface and typically include the results of quality control checking of Ribo-seq and uORF identifications. (**A**) The basic clean statistics of RPFs. (**B**) The RPF length distributions. (**C**) The RPF distribution on the coding frame (frames 0, 1 and 2). (**D**) 3-nt periodicity checking based on RPF metagene profile (added by P-site offset) on 5′ UTR and CDS start region. (**E**) A table containing the uORF ID, uORF position in the genome, uORF type, start codon, transcript ID, transcript start, transcript end, and link to RiboMeta tool to show the metagene distribution of RPFs on transcripts.

In the current database, we only include uORFs from publicly available Ribo-seq datasets. Researchers wishing to analyze uORFs can use uORFscan, available from the ‘Tools’ menu, to upload and decode their Ribo-seq datasets (Figure 4). uORFscan is developed based on the integrated pipeline for Ribo-seq data analysis mentioned in ‘Data analysis pipeline’ section and the main functions include sequencing quality controls, Ribo-seq quality evaluations, and uORF calling and annotations. To accelerate uploading, we recommend that datasets are converted into collapsed FASTA format or further compressed into zip or gz formats. The header in the collapsed FASTA file should appear as follows: ‘>seq1_x290’, where ‘seq1’ is a user-definable unique ID, and ‘290’ represents the RPF count of ‘seq1’. We have provided a Perl script to convert files into collapsed FASTA format on GitHub (https://github.com/rnainformatics/ribo-uORF). The maximum allowed size of each upload is 1 Gb. Once processing is completed, users can retrieve the results with the job ID (a string with 16 characters). uORFscan includes several user-adjustable parameters. For example, length interval can be set in advance, and only RPF sequences within this interval (27–32 nt by default) will be considered in downstream analyses. Other useful parameters include number of allowed mismatches (2 by default), maximum of multiple-mapping times (unique mapping by default) during RPF sequence mapping, unique molecular identifier (UMI) for PCR duplication elimination, and uORF score (0.5 by default) for uORF calling. For Ribo-seq data with UMI, the methods implemented in RiboFlow-RiboR-RiboPy (72) and UMI-Reducer (https://github.com/smangul1/UMI-Reducer) are used to remove the UMI and PCR duplication in uORFscan. Correct Ribo-seq inputs and suitable parameters are suggested on the webpage as an example to the user. Finally, we developed a ‘Retrieve results’ module to allow users to query the job status and download the analyzed results by searching job IDs.

All uORFscan outputs are presented via an intuitive web interface (Figure 4) and typically include the results of quality control checking of Ribo-seq and uORF identifications. Three main output functions are available. First, basic statistics on RPFs, including clean RPFs, can be obtained by mapping different potential contaminating-RNA types (rRNAs, tRNAs, and snRNAs) using Bowtie v1.2.2 (43) (Figure 4A). RPF mapping statistics on genome (mapped RPF and non-mapped RPFs) (Figure 4A) and RPF length distributions (Figure 4B) can also be generated. Second, users can evaluate the quality of Ribo-seq data according to the RPF distribution on the coding frame (frames 0, 1 and 2) (Figure 4C), RPF metagene profile representing the average quantitative ribosome density of all mRNAs, and a periodicity plot based on RPF distributions (added by the P-site offset) (Figure 4D). Third, uORFs uncovered using the same method we used to identify uORFs from publicly available Ribo-seq datasets can be presented in a table containing the uORF ID, uORF position in the genome, uORF type, start codon, transcript ID, transcript start, transcript end and link to RiboMeta tool to show the metagene distribution of RPFs on transcripts (Figure 4E). For each interactive plot, uORFscan provides links (at the top of the plot) for downloading the plot data in both txt and csv formats. Moreover, users can download all results presented in tables and figures using the ‘Download the results’ button found on each results page.

Although evidence is increasing that mutations within uORFs are linked to many diseases (21–24), an easy-to-use, integrated tool to annotate variations in 5′ UTRs is still lacking. We therefore developed UTR5var, a convenient tool based on UTRannotator (73), to allow users to upload variations files in VCF format and evaluate the effects of mutation on uORF functions for human (hg38) and mouse (mm10) uORFs (Supplementary Figure S4A). UTR5var first extracts all variations on 5′ UTR from the input VCF file and then annotates these variations using UTRannotator. The results from UTR5var are output via an intuitive web interface (Supplementary Figure S4B). Variations are classified according to their effects on uORFs (affecting or non-affecting) based on whether the position of the variation overlaps with any uORFs and also the variation effect. Variation effects are annotated using vep v100.3 (74) and are shown as a pie chart. Furthermore, the distribution of nucleotide changes affecting uORFs are counted and presented as a bar plot, and details on variations affecting uORFs are listed in a table that includes the variation ID, nucleotide changes, chromosomal position, Gene ID, transcript ID, and variation effect. The results output from UTR5var, including data tables and high-quality plots, can be downloaded by clicking the ‘Download’ button (at the top of the plot).

## EXAMPLE APPLICATIONS

**Figure 5.**
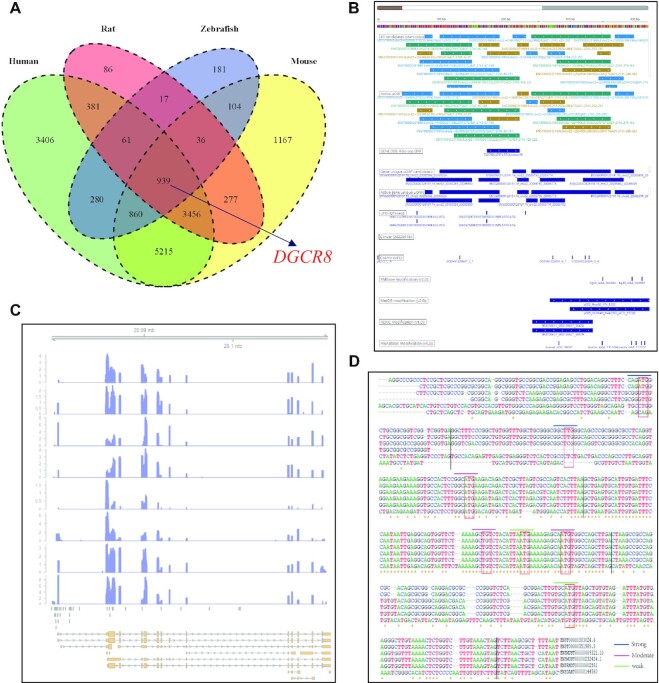
uORFs are widely distributed and strongly conserved in *DGCR8* among human, mouse, rat, and zebrafish. (**A**) Comparison of genes with uORFs among human, mouse, rat and zebrafish. (**B**) Overview of human *DGCR8* uORFs and other related information in mRNAbrowse. (**C**) RPF distribution of *DGCR8* uORF in IGV based on the BAM mapping file. (**D**) sequence conservation analysis of uORFs in *DGCR8* based on multiple sequence alignment between different species.

*DGCR8*, a component of the Microprocessor complex, acts as an RNA- and heme-binding protein involved in the initial step of microRNA biogenesis (75,76). To investigate 5′ UTR-mediated posttranscriptional control of *DGCR8* expression (77), we explored the uORFs and *cis*-elements of *DGCR8* in human, mouse, rat, zebrafish, fruit fly, and worm. A total of 939 genes with uORFs in the Ribo-uORF database were found to be common among human, mouse, rat, and zebrafish (Figure 5A). *DGCR8* possesses 25 uORF candidates, with 18 actively translated uORFs identified from 179 human Ribo-seq samples (Figure 5B). We also found that uORFs of *DGCR8* are associated with human diseases and RNA modifications (Figure 5B). As revealed by QTI-seq, uORFs of *DGCR8* generally use ATG as a start triplet (Figure 5B). We also checked the distribution of RPFs of *DGCR8* in IGV (78) based on the BAM mapping file. Intriguingly, we captured uORF signals from the 5′ UTRs of *DGCR8* in human, mouse, rat, and zebrafish (Figure 5). Consistent with this result, Kozak analysis based on multiple sequence alignment between different species suggested that Kozak consensus sequences are widely present in the 5′ UTR of *DGCR8* (Figure 5D). These results indicate that uORFs may influence *DGCR8* translation and suggest the possibility of experimentally manipulating levels of *DGCR8* expression at the translational level by editing the 5′ UTR of this gene.

## COMPARISON WITH OTHER RESOURCES

Several databases containing uORF information on distinct species were already established prior to the development of Ribo-uORF (Table 1). For instance, uORFdb was manually curated from uORF-related literature listed in the PubMed database (38), and TISdb was developed to house alternative translation initiation sites based on QTI-seq data from human and mouse (39). sORFs.org v2.0 is a public resource of small open reading frames (sORFs) identified by Ribo-seq (40). PsORF was specifically developed for plant sORFs based on multi-omic datasets, including genome, transcriptome, Ribo-seq, and MS data from public databases (79). uORFlight integrates potential uORF-, genetic variation- and uORF-related literature to enable the formulation of hypotheses of uORF functions in plant development or adaption to changing environments (80). SmProt v2.0 contains records of small proteins encoded by genes, especially those from UTRs and non-coding RNAs (41). Phase I Ribo-seq ORFs are available in a consolidated catalog of ORFs from seven publications annotated with GENCODE v35, but they cannot be browsed or visualized on the existing web server (50). RPFdb v2.0, a database of genome-wide information on translated mRNAs generated by Ribo-seq, only provides genomic coordinate information for uORFs (81). OpenProt v1.3 contains the possible ORFs longer than 30 codons across 10 species and integrates supporting evidence such as translation and expression (82). In the present study, we integrated uORF datasets from GENCODE Ribo-seq ORFs (50), TISdb (39), sORFs.org v2.0 (40) and uORFdb (38) into our Ribo-uORF database. Compared with current uORF-related databases, Ribo-uORF possesses several advantages: (i) uORF-specific development; (ii) a more comprehensive collection, including Ribo-seq and QTI-seq data; (iii) integration of other available resources, including disease/trait-associated SNPs, eQTLs, and RNA editing, RNA modification, and RBP binding information and (iv) supported transcript and genome browsers, peptide sequence viewer and Ribo-seq metagene visualizations.

**Table 1.** Comparison of Ribo-uORF with other uORF related resources

Name	Ribo-uORF	OpenProt v1.3	Trips-Viz	uORFlight	uORFdb	TISdb	sORFs.org v2.0	PsORF	SmProt v2.0	RPFdb v2.0	GENCODE ORFs
Type	Complex database	Complex database	Complex database	Database	Database	Database	Database	Database	Database	Database	Dataset
Data resources:											
Number of species	6	10	10	17	13	2	6	35	8	33	1
Ribo-seq	1495	87	2651	No	No	No	78	103	419	3603	139
QTI-seq	77	No	No	No	No	5	Yes	No	No	No	na
Public related resources	18	Yes	No	Yes	Yes	Yes	Yes	Yes	Yes	No	na
Tools:											
Supporting user-loaded datasets	Yes	Yes	Yes	na	na	na	na	na	na	na	na
Quality controls for user's data	Yes	Yes	Yes	na	na	na	na	na	na	na	na
uORF calling and annotations for user's data	Yes	Yes	Yes	na	na	na	na	na	na	na	na
Variation effects on uORF	Yes	No	No	na	na	na	na	na	Yes	na	na
Visualizations:											
mRNA coordinates	Yes	No	Yes	No	Yes	Yes	No	No	No	No	na
Genomic coordinates	Yes	Yes	No	No	Yes	Yes	No	Yes	Yes	Yes	na
Peptide visualizations	Yes	Yes	No	No	No	No	Yes	Yes	Yes	No	na
Ribo-seq metagenes	Yes	No	Yes	No	No	No	No	No	No	No	na
The ways to query datasets:											
Gene ID	Yes	Yes	Yes	Yes	Yes	Yes	No	Yes	Yes	Yes	na
Transcript ID	Yes	Yes	Yes	Yes	Yes	Yes	Yes	Yes	Yes	Yes	na
Sample accession	Yes	No	No	No	No	No	Yes	No	Yes	Yes	na
Available at	http://rnainformatics.org.cn/RiboUORF	https://openprot.org/	https://trips.ucc.ie	http://www.rnairport.com:443	https://www.bioinformatics.uni-muenster.de/tools/uorfdb/	http://tisdb.human.cornell.edu	http://sorfs.org	http://psorf.whu.edu.cn	http://bigdata.ibp.ac.cn/SmProt	http://sysbio.gzzoc.com/rpfdb/index.html	https://www.gencodegenes.org/pages/riboseq_orfs

‘na’ indicates that this feature of according resource is not applicable for comparison.

There are many existing tools for ORF calling from Ribo-seq data, such as RibORF (47), but these typically require many bioinformatics expertise to install, configure and manipulate. Ribo-uORF provides a convenient and intuitive one-stop web-based tool that supports Ribo-seq quality evaluation, uORF calling and annotation from Ribo-seq data. Besides, Ribo-uORF allows variation effect annotations on uORFs from user-uploaded variations. Trips-Viz provides visualizations for many processed Ribo-Seq and RNA-seq data in their database and supports ORFs detection for user-loaded Ribo-seq and RNA-seq data (83). However, an account is needed to upload user's data in Trips-Viz. OpenProt supports ORF detection from user-loaded mass spectrometry and Ribo-seq data. However, OpenProt requires that the data must be available in the public databases, including PRIDE Archive for MS data and Gene Omnibus Archive for Ribo-seq data (82).

## CONCLUSIONS AND FUTURE PERSPECTIVES

Recent advances in Ribo-seq have greatly facilitated efforts to decipher uORFs at the transcriptome-wide level (19). Ribo-seq and genome editing experiments have established that uORFs regulate the translational efficiencies of downstream mORFs (84,85). Despite the growing awareness of the widespread importance of uORFs in translational control of gene expression, a comprehensive resource for uORF annotation and characterization is currently lacking. Ribo-uORF now supports 501 554 uORFs generated from 1495 Ribo-seq datasets of six species (human, mouse, zebrafish, fruit fly, and worm) and 107 914 uTISs from 77 human and mouse QTI-seq datasets. We have also associated uORFs with an extra 18 related datasets from public databases, such as the GWAS Catalog (52) and REPIC (58). User-friendly browsers with genomic or transcript coordinates are provided to facilitate the exploration of uORFs. Moreover, uORFscan and UTR5var have been devised to support uORF analysis of user-uploaded data. We will continue to periodically update Ribo-uORF with new datasets and add additional species as more Ribo-seq and QTI-seq datasets become available. uORFscan and UTR5var will be fine-tuned and optimized to increase the amount of processable data. To facilitate downstream analyses, such as a translational efficiency analysis, we will continue to combine RNA-seq and Ribo-seq datasets in our database. We plan to maintain Ribo-uORF to ensure that this database continues to be a valuable resource for the research community.

## DATA AVAILABILITY

Ribo-uORF is freely accessible at http://rnainformatics.org.cn/RiboUORF and http://rnabioinfor.tch.harvard.edu/RiboUORF. Source codes for modified RibORF and related programs are available at https://github.com/rnainformatics/ribo-uORF.

## Supplementary Material

gkac1094_Supplemental_Files
