# Supplementary material for: Ribo-uORF: a comprehensive data resource of upstream open reading frames (uORFs) based on ribosome profiling
Source: Nucleic Acids Res. 2022 Nov 28;51(D1):D248–61. doi: 10.1093/nar/gkac1094 (PMC9825487; doi:10.1093/nar/gkac1094)
Supplement: gkac1094_Supplemental_Files [file gkac1094_supplemental_files.zip › Supplement_figures_and_table_S1_caption_all_in_one.pdf]

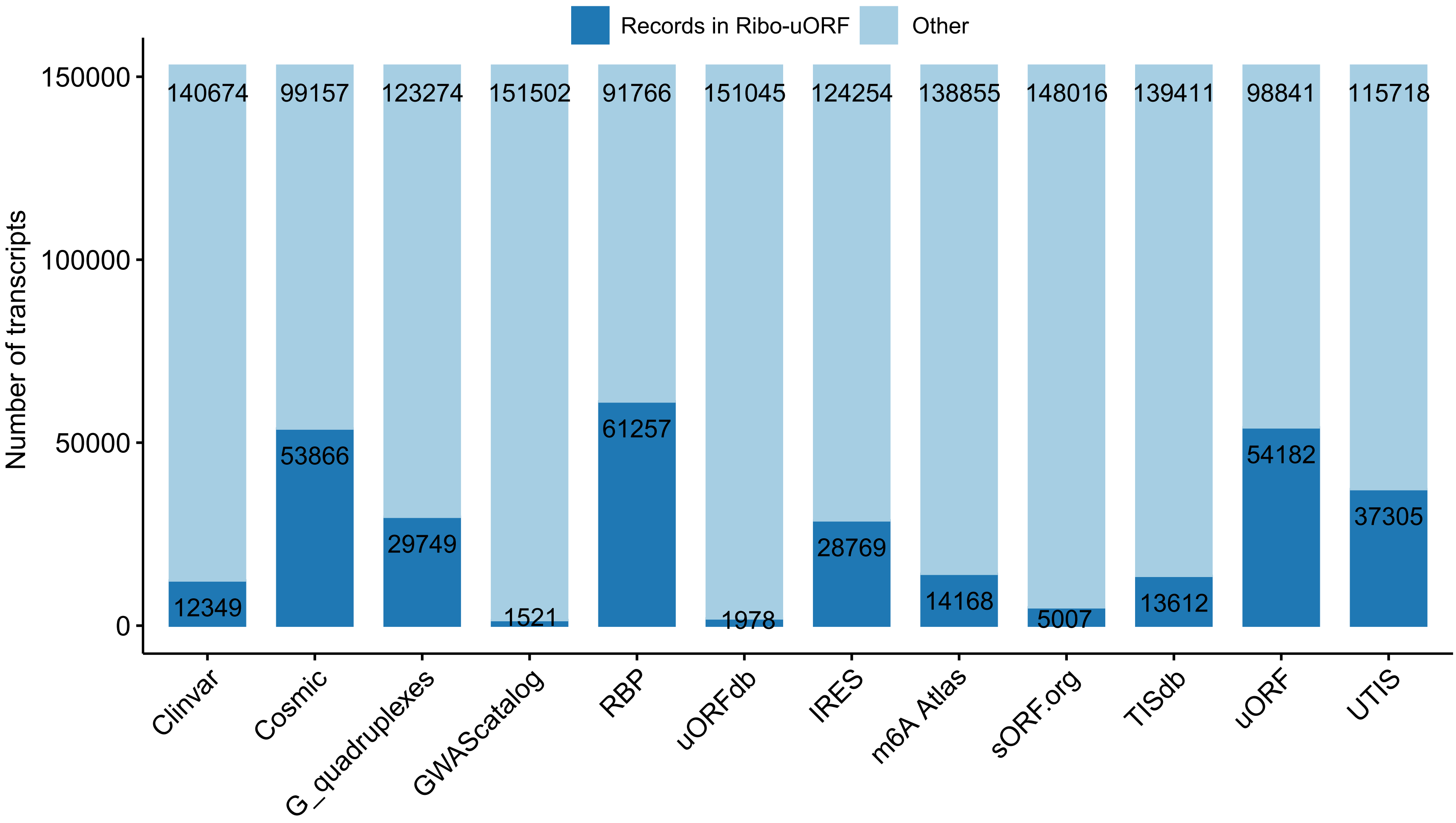

Figure S1. Statistics of different public annotation datasets from human in Ribo-uORF.

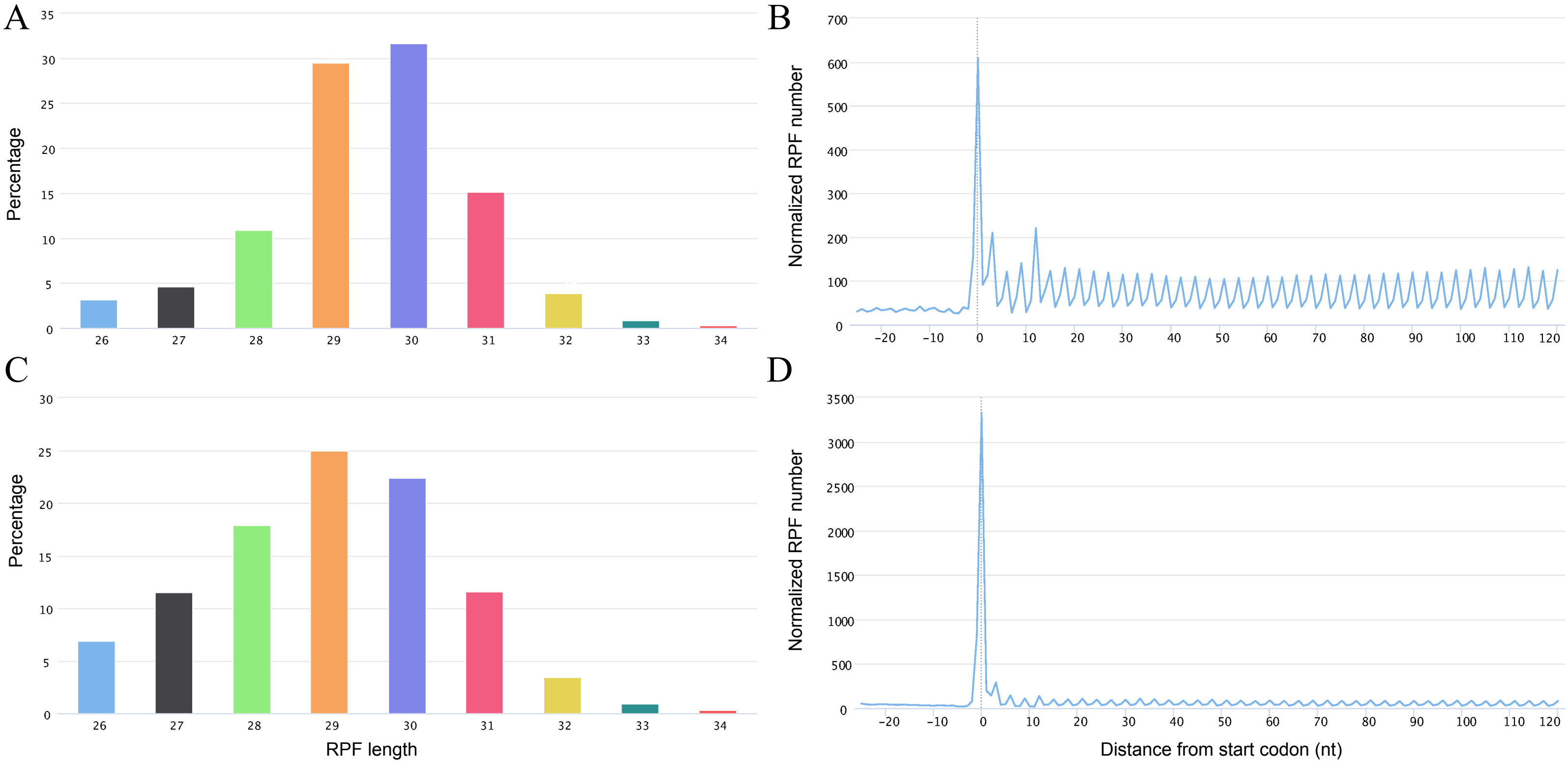

Figure S2. Examples of quality control of Ribo-seq and QTI-seq data. (A) and (B) Length and metagene distribution of RPFs generated by Ribo-seq. (C) and (D) Length and metagene distribution of RPFs generated by QTI-seq.

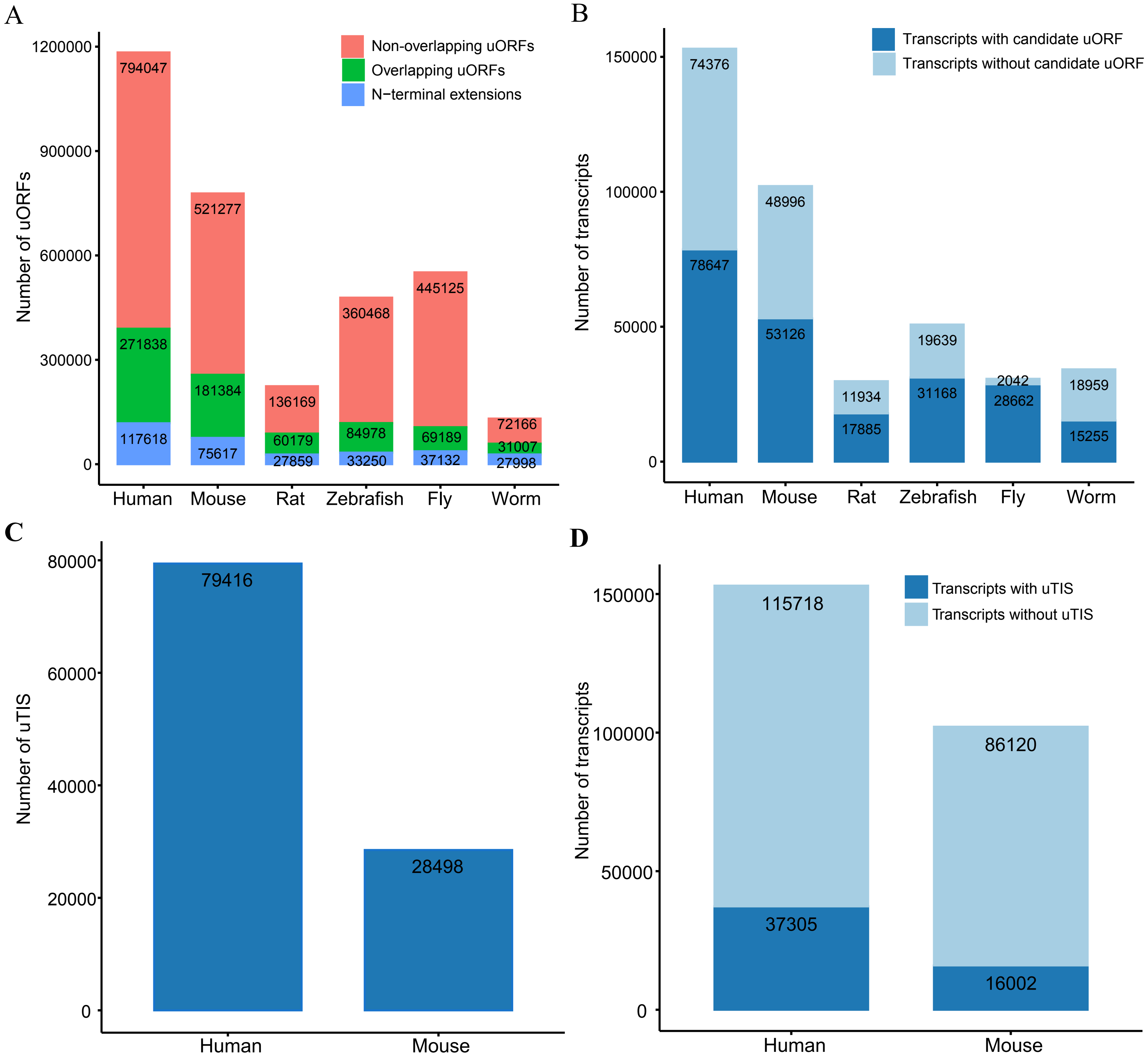

Figure S3. Statistics on candidate uORFs from six species. (A) Statistics on candidate uORF types. Non-overlapping, overlapping, and N-terminal extensions respectively refer to non-overlapping uORFs, out-frame overlapping uORFs, and N-terminal extension uORFs. (B) The number of transcripts with or without candidate uORFs. (C) The number of uTIS identified by QTI-seq in human and mouse, respectively. (D) The number of transcripts with or without uTIS generated by QTI-seq.

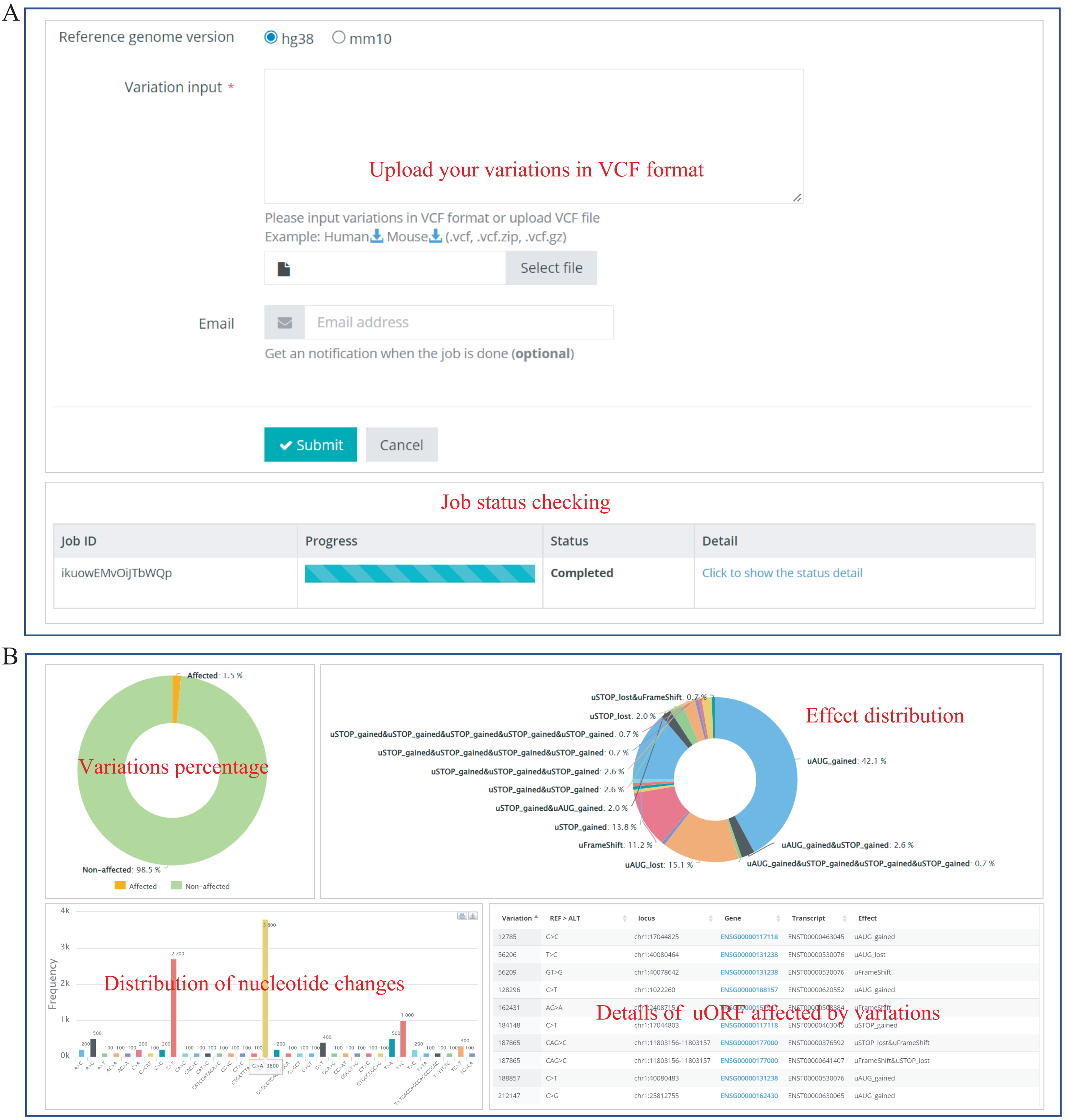

Figure S4. Screenshots of UTR5var. (A) uORFtools uses variations files in VCF format as input. The 'Retrieve results' module allows users to query the job status and download the analyzed results by searching job IDs. (B) The results from UTR5var are output via an intuitive web interface, which include variation classification (affecting or non-affecting), variation effects, the distribution of nucleotide changes affecting uORFs, and details on variations affecting uORFs that includes the variation ID, nucleotide changes, chromosomal position, Gene ID, transcript ID, and variation effect.

Table S1. Information on Ribo-seq and QTI-seq samples used in the Ribo-uORF database.
